# ISMB 2003 BioPathways SIG and 5th BioPathways Meeting

**DOI:** 10.1002/cfg.348

**Published:** 2003-12

**Authors:** Vincent Schachter, Aviv Regev

**Affiliations:** 1 GENOSCOPE 2 rue Gaston Crémieux Evry F-91000 France; 2 Bauer Center for Genomics Research Harvard University 7 Divinity Avenue Cambridge MA 02138 USA


**Comparative and Functional Genomics**
*Comp Funct Genom* 2003; **4**: 660–662.
Published online in Wiley InterScience (www.interscience.wiley.com). **DOI:** 10.1002/cfg.348

**Meeting Report**

### ISMB 2003 BioPathways SIG and 5th BioPathways Meeting

ISMB'03, Brisbane, Australia, 27–28 June 2003

Vincent Schachter[1]* and Aviv Regev[2]
[1] *Genoscope, 2 rue Gaston Crémieux, F-91000 Evry, France*
[2] *Bauer Center for Genomics Research, Harvard University, 7 Divinity Avenue, Cambridge, MA 02138, USA*

**Correspondence to:*
*Vincent Schachter, Director of Bioinformatics, GENOSCOPE, 2 rue Gaston Crémieux, F-91000 Evry, France.*
*E-mail: vs@genoscope.cns.fr*

Received: 10 October 2003
Accepted: 10 October 2003

The 5th BioPathways Consortium Meeting gathered 21 speakers, close to 100 registered participants and an undetermined number of visitors from neighbouring SIGs.

The meeting featured two main scientific sessions, focusing respectively on 'Regulation and Interactions on a Systems Scale' and 'Function and Evolution of Metabolic Networks', an 'Ontologies, Databases and Data Integration' session, and a contributed session on software tools for pathways. Following the BioPathways tradition and to foster depth of exchange, scientific sessions were structured as a series of long presentations, concluded by an hour of open discussion on the session theme.

The meeting started with a short assessment of the evolution of the field — computational biology of networks, or 'systems biology'? — which has matured fast in the 3 years of existence of the BioPathways SIG. While some theoretical subfields, such as network reconstruction from experimental data, are acquiring technical depth and generating predictions of increasing biological relevance, there is a clear trend towards a stronger coupling between theoretical and experimental approaches, leading to new open questions on both sides. Another noticeable trend is the strong revival of fields that had been perceived as fairly well understood and stable, such as metabolism, thanks both to the 'systems-wide' perspective and to new theoretical tools.

The 'Regulation and Interactions' session revolved around a few key ideas, each illustrated by several speakers. A first theme was the search for the right notion of 'module' in biological networks, at different levels of molecular organization (e.g. in regulatory networks, protein interaction networks), using diverse theoretical tools (e.g. graph theory, graphical probabilistic models).

Segal illustrated this theme by presenting a method to infer module networks, i.e. sets of genes sharing a regulatory mechanism, from expression data. The original inference scheme learns a Bayesian Network from the data, but the Bayesian network models regulation of sets of genes — modules — rather than single genes. The learning algorithm optimizes on the structure of the network, but also on the partition of genes into modules and on the 'regulation program' of each module, an abstract representation of the regulatory mechanism as a decision tree.

It was also shown that in order to ensure statistical robustness and, better yet, biological relevance,

Copyright © 2003 John Wiley & Sons, Ltd.

integration across different levels could be necessary. Searching for modules across different species was proposed as a promising path to increase support from experimental data, to filter out noise and perhaps to search for evolutionarily-driven design principles.

In an extension of the classical expression profile correlation and clustering approaches, Segal showed how microarray data from several species could be combined to yield a 'meta-gene' network. First, genes are grouped across species into so-called 'metagenes' (groups of orthologues), using an extension to *n* species of the BLAST bidirectional best hit criterion. Expression profiles are then transformed into gene–gene correlation matrices for each species, and these are merged into a single metagene–metagene correlation matrix. Discretization yields a metagene 'co-expression network', which was then subjected to a variety of statistical analyses, and used to predict function using guilt-by-association. Among the biological insights that could be gained, the assessment of the fraction of co-expression links conserved from the single to multi-species level showed clear structure in functional space: links between genes related to metabolism, degradation and protein biosynthesis were among the most conserved, while signalling and neuronal function seemed to be newly evolved.

Another theme was the use of stochastic models to better account for molecular phenomena for which ordinary or partial differential equations modelling is inadequate, and of stochastic inference methods. Tian showed how stochasticity arises naturally in regulatory networks, from the probabilistic nature of the binding process combined with the low number of transcription factors present at a given time. He also surveyed how noise could be of functional use to the cell, by helping it stabilize its dynamics or by even by generating qualitative differences in phenotype. Finally, he reviewed how existing types of network models, from Boolean circuits to models based on stochastic differential equations, could account for stochasticity in simulations.

A third theme was the use of probabilistic methods for the prediction of individual properties of genes and proteins, using sets of (other) properties. Przulj presented a systematic search for correlation between local graph properties of protein networks — including degree, articulation points, hubs, existence of short paths between vertices — and biological properties of the corresponding proteins, such as lethality, functional class, or the existence of a genetic interaction. Lichtenberg and Zhang also illustrated this theme, with respective focuses on protein function prediction and on genetic and protein interaction prediction.

Finally, both the issue of the robustness of predictive methods relative to false positives and negatives in experimental data, and the question of how to validate these predictions — from cross-validation to actual experiments — were ubiquitous. Although there appears to be no simple recipe to solve the validation issue, the level of sophistication of statistical assessments of prediction accuracy has risen, with combinations of strategies, such as systematic validation against functional categories, cross-correlation between different datatypes, or comparison against randomized networks.

Regarding the robustness issue, the common strategy is to increase support for predictions by pooling experimental information across several genes (modules, families), across species, and across types of experiments. As a consequence, predictions are generated on more abstract entities. The use of model-based statistical methods also facilitates the definition of optimality criteria for a model instance inferred from a given dataset, ensuring some measure of theoretical confidence in the predictions.

The open discussion addressed these four themes in detail, with a special focus on protein interaction prediction, for which several participants proposed the establishment of a comparative assessment of structure prediction (CASP)-like competition.

The 'Metabolic Networks' session illustrated well how the generation of measurements on a whole-cell scale is driving the need for models (static and dynamic) for network reconstruction methods and for analytical approaches.

An ambitious Japanese project to build a comprehensive global picture of *Escherichia coli* metabolism, within the larger context of the International *E. coli* Alliance, was described by Tomita. The project integrates several complementary ongoing experimental efforts: MS analyses of metabolites, *in vivo* dynamic analysis with labelled isotopes, expression profiling, 2D gels, systematic mutagenesis, enzyme kinetics, protein interaction identification, etc. For instance, 10 000 protein–protein interactions have been detected so far

Copyright © 2003 John Wiley & Sons, Ltd. 

using his-tagged proteins and immunoprecipitation, and a complete single-deletion mutant library has been generated. Another effort focuses on finding as-yet unidentified metabolites using CE/MS measurements for charged molecules and LC/MS for neutral ones, the combination allowing a good resolution on the identification of peaks of distinct metabolites. First results show a surprisingly large number of metabolites that are not present in existing databases. Tomita confirmed that data generated by these projects will be made publicly available.

On the theoretical side, one recurring theme was the use of comparative approaches, e.g. to fill in the gaps in the reconstruction of a static metabolic network, or to explain major phenotypic differences between genetically close species using steady-state dynamics.

Shah presented an exploratory study aimed at reconstructing a minimal yet maximally self-sufficient metabolic network from KEGG multi-species metabolic data. In such a network, all enzymes necessary for survival with minimal input should be included, the goal being to connect every compound to the network, possibly with the help of as-yet unknown reactions that could perhaps be predicted. A first attempt generated a large number of hypotheses in need of biological validation.

Another important idea was that, whereas full dynamical models are difficult to both reconstruct from available data and to study, there are simplified models (flux balance analysis, S-systems, coupling of steady-state with small differential equations, etc.) based on specific biological hypotheses (e.g. steady-state, or proximity to such) which lend themselves better to simulation or analysis of network properties.

Martinos dos Santos showed how flux balance analysis (FBA), the study of metabolic fluxes under a steady-state hypothesis, can help understand why only one of two bacteria — *Pseudomonas putida* and *Ps. aeruginosa* — that are very similar genetically is pathogenic. FBA allowed the prediction that the presence of two enzymes specific to *Ps. aeruginosa* in the metabolic network has a significant influence on metabolic flux distribution. For instance, ATP production was increased in some extreme pathways, the minimal set of flows within the network from which all actual flux distributions can be derived as linear combinations and which define the maximum metabolite conversion capabilities of the network under a given set of input conditions. One lesson from this work is that small genetic differences can be strongly amplified.

Dos Santos then presented a modelling approach adapted to the study of metabolic fluxes in a community of interacting cells — typically bacteria. The approach combines FBA, yielding distribution of fluxes at each time point, with more detailed dynamic modelling at certain critical points.

Almeida surveyed existing mathematical models for metabolic networks, focusing on the feasibility of instantiating a model at a given level of detail using the metabolic profiles time series available today. Next, he presented S-systems, a dynamic model in which parameters have clear biological meaning, yet simplified enough to permit network reconstruction.

The discussion opened with the question of how much is actually known about metabolism. Differences between 'potential' and actually occurring pathways, or between canonical pathways and condition-specific pathways, were underlined. The main issue related to experimental data was, unsurprisingly, that of availability, followed closely by reliability. Several biases related to experimental conditions classical in the study of bacterial metabolism were also discussed.

The 'Databases and Ontologies' session featured presentations on pathways exchange formats and languages by a fairly comprehensive selection of the groups working towards a standardization goal (SBML, BioPax, CellML, OMG-LSR), as well as presentations on pathways databases, for which there is clearly an unfilled need. In particular, Luciano described recent and impressive progress in the BioPax effort to define a standard format for pathways exchange, and Hucka gave an update on the evolution and adoption of the SBML (systems biology mark-up language) format by modellers.

Finally, the 'Software Tools' session included presentations on a variety of pathways data management and visualization tools.

In summary, the meeting confirmed that the field of networks-related computational biology is more than ever in a fast-growth stage, with both frontier and depth expanding, and provided a snapshot of that field. Next year's meeting in Glasgow will undoubtedly photograph a fairly different landscape. More details of the meeting can be found at **www.biopathways.org**

Copyright © 2003 John Wiley & Sons, Ltd.

